# Electrocardiographic Effects of Bupropion Toxicity Suggesting Dysfunction of the Gap Junction or Connexin 43

**DOI:** 10.7759/cureus.56288

**Published:** 2024-03-16

**Authors:** Patrick Bruss, Ryan Hartle, Jennifer Astacio, Ammar F Chauhdri

**Affiliations:** 1 Emergency Medicine, ProMedica Monroe Regional Hospital, Monroe, USA; 2 Emergency Medicine, University of Michigan, Ann Arbor, USA

**Keywords:** drug overdose and poisoning, ecg, gap junction toxicity, connexin-43, bupropion

## Abstract

This is a case of a 20-year-old pregnant female presenting EKG abnormalities associated with an overdose of bupropion. These ECG abnormalities are prolongation of the QRS, prolongation of the corrected QT interval (QTc), right axis deviation, and a terminal R wave. The propagation of electricity through the myocardium is dependent on many factors. It is dependent on the flow of sodium from the extracellular to intracellular space, flow of potassium from intracellular to extracellular space, and ultimately the propagation of the signal at the gap junction by Connexin 43 (Cx-43). We postulate that the ECG abnormalities in this case are secondary to bupropion's effect on the potassium rectifier channels (Kir) and or Cx-43 at the gap junction.

## Introduction

Bupropion is a norepinephrine-dopamine reuptake inhibitor used mainly in the treatment of depression but may also be used in the treatment of smoking cessation. Bupropion toxicity is a potentially lethal toxidrome if >450 mg is ingested [1]. Potential patient presentation includes but is not limited to agitation, nausea, vomiting, tremor, tachycardia, seizures, hypotension, coma, and cardiac arrest. Treatment includes activated charcoal, benzodiazepines for seizures, vasopressors in cases of hemodynamic instability, sodium bicarbonate for prolonging QRS, and intralipid or extracorporeal membrane oxygenation (ECMO) for severe, refractory cases [2]. Sodium bicarbonate has not been definitively shown to narrow the QRS duration for bupropion overdose [3]. Disposition varies greatly as it primarily depends on hemodynamic stability and clinical picture after 8 hours. If the patient is asymptomatic after 8 hours, they may be discharged. Data suggests that inhibition of the potassium rectifier channels and or Cx-43 may result in cardiac arrhythmias [4-6].

We present the ECG findings of a pregnant 20-year-old female who presented to the ED after intentionally overdosing on bupropion. There were no other co-ingestants. The electrocardiogram (ECG) abnormalities for tricyclic antidepressant (TCA) toxicity are well known and described in medical literature, namely, right axis deviation (RAD), terminal R wave, prolongation of the QT interval, and QRS complex [7]. This patient demonstrated ECG abnormalities similar to those seen in TCA toxicity, as seen through serial ECGs collected throughout the patient’s care that demonstrated a wide complex tachycardia with a progressively prolonging corrected QT interval (QTc), RAD, and a terminal R wave.

This case provides an opportunity to discuss the potential mechanisms and management of drug interactions on electrical propagation. Therefore, we find that the ECG findings associated with TCA toxicity are not unique to TCA overdose, and could be seen in medications that affect the channels necessary for normal electrical propagation, such as bupropion [8]. We also propose the toxic effects of certain medications on the components that are vital in the propagation of electricity in the myocardium and the brain can result in primary or secondary gap junction toxicity (GJT). Further investigation is needed to develop more effective therapies for treating GJT.

## Case presentation

**Figure 1:**
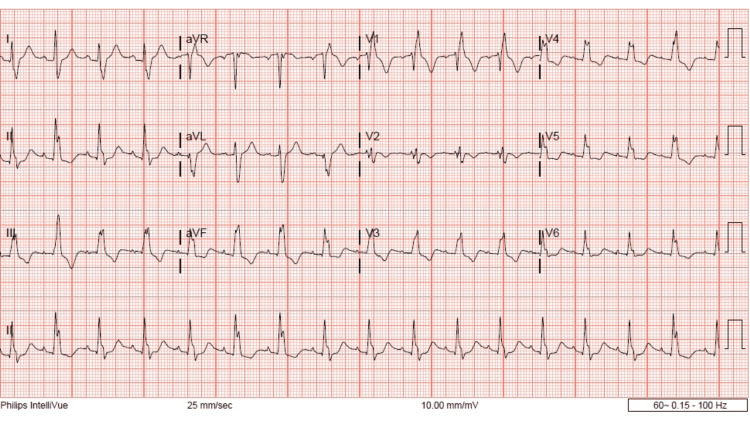
Day 1, initial ECG, 18:08 hours, impending RAD, LPFB, no terminal R wave aVR. RAD: right axis deviation; LPFB: left posterior fascicular block

A 20-year-old female, 11 weeks pregnant, with a past medical history of depression, and Tetralogy of Fallot with surgical repair as a child, presented to the ER after a suicide attempt. The patient reported that she ingested an unknown quantity and concentration of extended-release formulation bupropion tablets 30-35 minutes prior to arrival and denied ingestion of any other medication drug or alcohol. The patient estimated that there were approximately 30-40 pills in the bottle. On arrival, the patient complained of lightheadedness and near syncope. She denied any fever, chills, shortness of breath, chest pain, nausea, vomiting, abdominal pain, diarrhea, constipation, dizziness, or headache. Initially, the patient was resting in bed, in no acute distress. The patient was speaking in complete sentences, with normal phonation, no drooling, no audible wheezing/stridor, and no accessory muscle use. Vital signs were stable. Initial ECG showed sinus rhythm, right bundle-branch block (RBBB), left posterior fascicular block (LPFB), QRS 159 ms, QTc 493 ms, RAD, and no terminal R wave (Figure 1).

**Figure 2:**
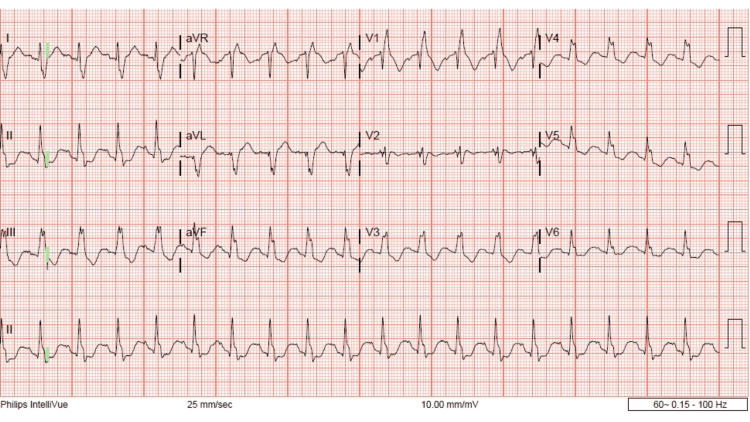
Day 1, second ECG, 19:15 hours, RAD, LPFB, terminal R wave aVR.

The patient was given activated charcoal due to the ingestion being within one hour of presentation. Poison control was contacted and advised that the patient be observed for any signs of dysrhythmias, agitation, or seizures. Poison control recommended treating the patient with supportive care and benzodiazepines as necessitated. Upon re-assessment, the patient was tachycardic at 120 beats per minute. She also complained of nausea and had vomited the activated charcoal. Patient was given 4 mg ondansetron and 1 mg of lorazepam. Repeat ECG as marked with green annotations showed sinus tachycardia with RAD, LPFB, terminal R wave, QRS 164 ms, and QTc 655 ms (Figure 2).

**Figure 3:**
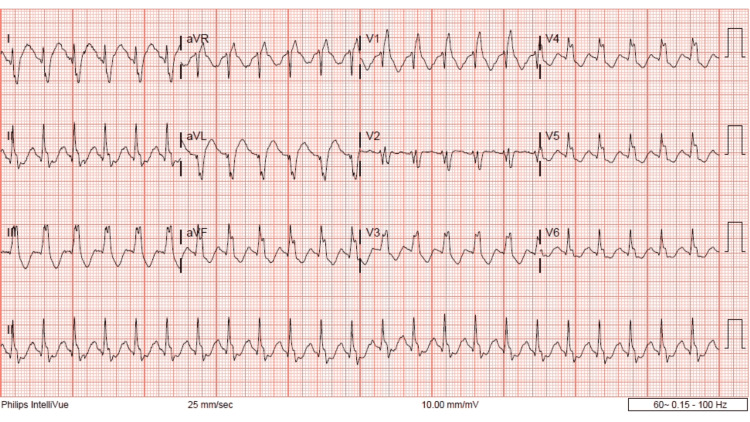
Day 1, third ECG, 20:08 hours, worsening RAD, LPFB, terminal R wave aVR. RAD: right axis deviation; LPFB: left posterior fascicular block

The patient reported on additional re-evaluation that she was no longer nauseous; however, she complained of paresthesias. A fluid bolus was then ordered and given. Upon re-evaluation within an hour of IV fluids, the patient was resting comfortably in bed. Her heart rate was 100 beats per minute and her blood pressure was within normal limits. Medical clearance labs were significant for mild hypokalemia of 3.4 mEq/L but were otherwise unremarkable. Upon additional reassessment, the patient's heart rate became tachycardic at 140 beats per minute. Repeat ECG again demonstrated sinus tachycardia with RAD, LPFB, terminal R wave, QRS 163 ms, and QTc 644 ms (Figure 3).

**Figure 4:**
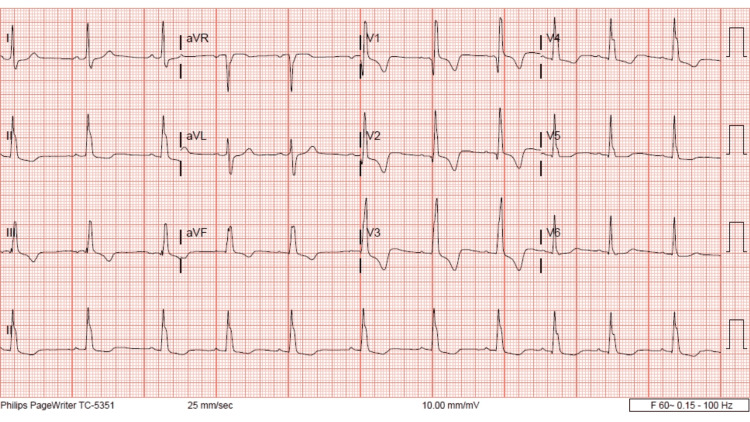
Day 4, last ECG during medical admission. Resolution of RAD and terminal R wave aVR. RAD: right axis deviation

The patient’s nurse then witnessed the patient actively seizing. Upon arrival at the bedside, the patient was not actively seizing; however, the patient was given an additional 2 mg lorazepam. Poison control was then updated, they again recommended benzodiazepines and supportive care. The toxicologist recommended keeping the potassium above 4 mEq/L, the magnesium above 2 mEq/L, and the calcium above 9.5 mEq/L. Further recommendations were to treat the patient with benzodiazepines and barbiturates as needed and to use propofol if intubation was necessitated. The patient was supplemented with magnesium, calcium, and potassium and was subsequently admitted to the ICU. Five days later the patient had a resolution of clinical symptoms and ECG findings (Figure 4).

## Discussion

Cardiac monocyte function, as well as the ECG appearance, is dependent on the ability of ion channels to propagate electricity through the myocytes and ultimately the gap junctions between the cardiac myocytes. Bupropion has a known effect on the K_ir_ channels of the heart [5] and can lead to QRS widening [9]. In this case, we see the QRS widening and QTc prolongation but we also see RAD and a terminal R wave as well. It is interesting to see these abnormalities which are usually associated with TCA toxicity secondary to this medication's effect on the cardiac sodium channels. This suggests that any medication or drug that affects any of the molecular elements necessary for normal signal propagation may result in similar ECG abnormalities.

There is literature that supports bupropion’s effect on the K_ir_ channels and Cx-43 at the gap junction [4-6,9,10]. This patient’s physical examination findings of agitation, somnolence, and seizure accompanied by widening QRS, prolongation of QT interval, RAD, and terminal R wave indicate that the main issue is the toxic effect on the K_ir_ channels in the myocardium and astrocytes since bupropion has minimal effect on the sodium channels [11]. However, since bupropion has been shown to inhibit Cx-43, it is also possible that bupropion's effects on Cx-43 are responsible for this patient's clinical presentation and ECG abnormalities [6,9,10]. This patient had both clinical and electrocardiographic signs of toxic effects on the components that are vital in the propagation of electricity in the myocardium and the brain. In this case, we cannot conclusively say which one of these elements is responsible. However, the propagation of electricity from cell to cell is ultimately dependent on the gap junction. Therefore, whether it's the connexin proteins at the gap junction itself or upstream from the gap junction via the sodium or potassium channels, we postulate the same ECG abnormalities (RAD, terminal R wave, QRS, and QT prolongation) may be present as was the case for this patient.

It has been reported that bupropion acts on gap junctions through the protein Cx-43 [6,10]. It is known that gap junctions are co-localizing with sodium channels in the myocardium, specifically intercalated disks, which are essential for cardiac conduction within the myocardium [12]. Furthermore, previous studies indicate that bupropion has been shown to affect calcium signaling in cells, which could in turn affect the activity of connexin-43. A study conducted by Lin et al. in 2011 demonstrated how bupropion suppressed Ca^2+^ channels to inhibit glutamate release in the cerebral cortex of rats [13]. And, while the mechanism is unknown at this time, one potential pathway in which this may occur (based on a 2007 study analyzing intracellular calcium regulation of Cx-43 in mice lens cells) is that bupropion may act as a noncompetitive inhibitor of connexin hemi channels, which are half of a gap junction channel that allows for the passage of small molecules and ions [14]. Additionally, there is evidence that the permeability of Cx-43 gap junctions is regulated by the cell’s intracellular calcium concentration [14,15]. Literature suggests that bupropion has a role as a calcium channel inhibitor, it can be speculated that the bupropion serves to increase intracellular Ca^2+^ concentration via the blocking of calcium-related hemi channels, thus causing inhibition and closure of Cx-43 gap junctions [15,16].

In a recent publication by Losso et al., they review the molecular effects of bupropion on Cx-43 [6]. They also propose that rotigaptide or danegaptide may be useful in the treatment of bupropion overdose via its effect on the phosphorylation of Cx-43. However, these medications have only been used in rat models during ischemia and their use in human patients is theoretical at this point.

This patient did have a history of Tetralogy of Fallot (TOF) repair as a child. This does result in abnormalities in her baseline ECG which we can see in Figure 4. Specifically an incomplete right bundle branch block, fractionated QRS, and nonspecific t-wave abnormalities. However, it is important to note that in her baseline ECG the QRS is less than 120 ms, this is no RAD and there is no terminal R wave. This indicates that any ECG changes for this patient were not related to her previous cardiac surgery. One could also make the argument that the prolongation of the QRS is secondary to a rate-dependent right bundle-branch block (RDRBBB). However, we do not feel that this is the case in this instance. There is literature about the electrophysiology behind a RDRBBB, and the majority of RDRBBB was observed at cycle lengths below 500 ms [17-19]. In Figure 1, the patient has a heart rate of 102 bmp and a cycle length (Cl) of 588 ms (60000/h), which is above 500 ms. Additionally, if the QRS prolongation was due to the heart rate one would expect the QRS to change proportionally with the heart rate but we do not see that here. In Figure 2, the heart rate is 114 bmp, the Cl is 526 ms and the QRS is 164 ms. In Figure 3, the heart rate increases to 138 bpm, the Cl decreases to 434 ms but the QRS remains similar at 163 ms. These facts, while not conclusive, do suggest that the QRS prolongation is due to bupropion.

## Conclusions

To our knowledge, this is the first case of a bupropion overdose with ECG abnormalities mimicking those seen in tricyclic antidepressant (TCA) toxicity, specifically QRS prolongation, QTc prolongation, as well as right axis deviation (RAD) and a terminal R wave, which appears to be unique. This suggests that these findings may not be unique to TCA overdose and could also be observed in medications like bupropion. Unfortunately, we were not able to follow-up with the patient after her discharge from the ICU or measure the potential impact of her pregnancy. We were able to identify and treat the patient's symptoms effectively, and the patient was able to make a full recovery. Furthermore, we could not determine long-term outcome data or assess the effectiveness of the proposed treatments, given the nature of this case. The patient's history of Tetralogy of Fallot adds complexity to the cardiac issues and makes determining the mechanism more challenging.
